# Supplementary material for: Enhanced tactile coding in rat neocortex under darkness
Source: eLife. 2026 Sep 21;14:RP106554. doi: 10.7554/eLife.106554 (PMC13592814; doi:10.7554/eLife.106554)
Supplement: Peer review history

# Editor report: eLife Assessment

**DOI:** 10.7554/eLife.106554.3.sa0

**Authors:** Julijana Gjorgjieva

**Affiliations:** Technical University of Munich Germany

**Keywords:** Solid, Valuable

This study presents **valuable** findings and employs modern analytical approaches on how transient absence of visual input (darkness) affects tactile encoding in the rat somatosensory cortex (S1). The evidence supporting the authors' claims is **solid**, as population-level neural activity recorded in S1 and decoded by a CNN carries more discriminable texture information in darkness. The underlying basis of this effect remains only partly resolved, however, because it is still unclear which neural features from the CNN drive the decoding and if visual interference is appropriately accounted for, which might confound true neural representational change.

# Referee report: Reviewer #1 (Public review)

**DOI:** 10.7554/eLife.106554.3.sa1

Summary:

The authors aimed to investigate how short-term visual deprivation influences tactile processing in the primary somatosensory cortex (S1) of sighted rats. They justify the study based on previous studies that have shown that long-term blindness can enhance tactile perception, and aim to investigate the change in neural representations underlying rapid, short-term cross-modal effects. The authors recorded local field potentials from S1 as rats encountered different tactile textures (smooth and rough sandpaper) under light and dark conditions. They used deep learning techniques to decode the neural signals and assess how tactile representations changed across the four different conditions. Their goal was to uncover whether the absence of visual cues leads to a rapid reorganization of tactile encoding in the brain.

Strengths:

The study effectively integrates high-density local field potential (LFP) recordings with convolutional neural network (CNN) analysis. This combination allows for decoding high-dimensional population-level signals, revealing changes in neural representations that traditional analyses (e.g., amplitude measures) failed to detect. The custom treadmill paradigm permits independent manipulation of visual and tactile inputs under stable locomotion conditions. Gait analysis confirms that motor behavior was consistent across conditions, strengthening the conclusion that neural changes are due to sensory input rather than movement artifacts.

Weaknesses:

(1) While the study interprets the emergence of more distinct texture representations in the dark as evidence of rapid cross-modal plasticity, the claim rests on correlational data from a short-term manipulation and decoding analysis. The authors show that CNN-derived feature embeddings cluster more clearly by texture in the dark, but this does not directly demonstrate plasticity in the classical sense (e.g., synaptic or circuit-level reorganization). The authors have noted this as a limitation and have clarified that the observed changes reflect functional reorganization rather than structural plasticity.

(2) Although gait was controlled, changes in arousal or exploratory behavior in light versus dark conditions might play a role in the observed neural differences. The authors have controlled for various factors in relation to locomotion, but future studies would benefit from more direct behavioural readouts of arousal states (e.g., via pupillometry or cortical state indicators).

(3) It should be noted that the time course of the observed changes (within 10 minutes) is quite rapid, and while intriguing, the study does not include direct evidence that the underlying circuits were reorganized-only that population-level signals become more discriminable. The authors have adequately discussed this as an avenue for more mechanistic future research.

(4) The authors have adequately discussed that, while these findings are consistent with somatotopy and context-dependent dynamics, they do not provide strong independent evidence for novel spatial or temporal organization.

(5) The authors have also discussed that, while the neural data suggest enhanced tactile representations, the study does not assess whether rats' actual tactile perception improved. Future studies including an assessment of a behavioral readout (e.g., discrimination accuracy), would be insightful.

(6) The authors' discussion about the implications for sensory rehabilitation, including Braille training and haptic feedback enhancement was a bit premature, but they have amended this, and it remains an interesting translational potential to be explored in future studies.

(7) While the CNN showed good performance, more transparent models (e.g., linear classifiers or dimensionality reduction) appear to not exceed chance level. The implications of this are that there is an underlying complex structure in the LFPs that has yet to be fully uncovered, on the mechanistic level. This would be important to push the findings forward in future studies.

Therefore, while the authors raise interesting hypotheses around rapid plasticity, somatotopic dynamics, and rehabilitation, the evidence for each is indirect. Stronger claims will require future causal experiments, behavioral readouts, and mechanistic specificity beyond what the current data provides. However, the work represents an interesting starting point to a more mechanistic understanding in the future.

# Referee report: Reviewer #2 (Public review)

**DOI:** 10.7554/eLife.106554.3.sa2

Summary:

Yamashiro et al. investigated how transient absence of visual input (i.e. darkness) impacts tactile neural encoding in the rat primary somatosensory cortex (S1). They recorded local field potentials (LFPs) using a 32-channel array implanted in forelimb and hindlimb primary somatosensory cortex while rats walked on smooth or rough textures under illuminated and dark conditions. Employing a convolutional neural network (CNN), they successfully decoded both texture and lighting conditions from the LFPs. The authors conclude that the subtle differences in LFP patterns underlie tactile representation surface roughness and become more distinct in darkness, suggesting a rapid cross-modal reorganization of the neural code for this sensory feature.

Strengths:

• The manuscript addresses a valuable question regarding how sensory cortices dynamically adapt to changes in sensory context.

• The use of machine learning (CNNs) enables the analysis to go beyond conventional amplitude-based metrics, potentially uncovering subtle but meaningful effects.

• The authors have substantially improved the manuscript with clearer figures, additional statistical analyses (including permutation tests and cross-validation), and greater methodological transparency.

Weaknesses:

• The new analyses (grand-average LFPs, correlation maps, wavelet decompositions, attribution-score correlations) improve transparency but do not yet clarify which specific neural features the CNN exploits, leaving the central interpretability question unresolved.

• A plausible alternative explanation for the increased discriminability in darkness remains insufficiently ruled out: visually driven activity in the light condition (e.g., ambient illumination changes or self-motion-induced visual input) could contaminate S1 LFPs and account for the effect without reflecting a true neural representational change.

• Behavioural and order controls have been improved but remain somewhat limited in sample size.

Overall assessment:

The revised manuscript is clearer, more transparent, and technically strengthened. However, the true nature of the signal changes underlying the observed differences in discriminability remains unclear, limiting the scientific strength of the conclusions. The possibility that visual interference contributes to the observed effects remains a plausible and untested alternative interpretation. Additional experiments or analyses quantifying visually evoked activity in S1 would be required to confirm the claim of genuine reorganization of neural representation depending on the illumination condition.

# Author comment: Author response

**DOI:** 10.7554/eLife.106554.3.sa3

**Authors:** Kotaro Yamashiro, Tanaka Shiyori, Nobuyoshi Matsumoto, Yuji Ikegaya

**Affiliations:** The University of Tokyo Tokyo Japan; The University of Tokyo Tokyo Japan; The University of Tokyo Tokyo Japan; The University of Tokyo Tokyo Japan

The following is the authors’ response to the original reviews.

**Public Reviews:**

**Reviewer #1 (Public review):**

(1) While the study interprets the emergence of more distinct texture representations in the dark as evidence of rapid cross-modal plasticity, the claim rests on correlational data from a short-term manipulation and decoding analysis. The authors show that CNN-derived feature embeddings cluster more clearly by texture in the dark, but this does not directly demonstrate plasticity in the classical sense (e.g., synaptic or circuit-level reorganization).

Thank you for this insightful comment. We acknowledge that our claim of “rapid cross-modal plasticity” is based on correlational evidence and does not directly address synaptic or circuit-level reorganization, which would require more invasive methods. Our study instead focuses on changes in the representational structure of tactile stimuli when visual input is temporarily removed, highlighting the adaptability of sensory coding to environmental context. We agree that this distinction is important and have revised the manuscript to clarify that the observed changes reflect functional reorganization rather than structural plasticity, as indicated by the enhanced separability of texture representations in S1 during darkness.

(2) Although gait was controlled, changes in arousal or exploratory behavior in light versus dark conditions might contribute to the observed neural differences. These factors are acknowledged but not directly measured (e.g., via pupillometry or cortical state indicators).

Thank you for your insightful comment. We agree that arousal and exploratory behavior could influence neural differences and have considered these factors in our study. While gait was controlled, we did not directly measure arousal (e.g., via pupillometry or cortical indicators).

To partially address this, we reviewed locomotor-speed traces (Supplementary Figure 1), which showed no significant differences between light and dark conditions, suggesting movement speed did not drive the neural differences. We also reversed the order of light and dark conditions, and although the separability of textures was not significantly different, it further supports that motivation did not confound our results.

However, we acknowledge that arousal may still affect cortical dynamics, especially in the dark condition, where the lack of visual input might alter exploratory behavior. Due to technical limitations, we could not directly measure arousal states, and this is now discussed in the revised manuscript. While we cannot rule out the influence of arousal, the enhanced separability of texture representations suggests that sensory reorganization due to visual deprivation likely played a substantial role.

(3) Moreover, the time course of the observed changes (within 10 minutes) is quite rapid, and while intriguing, the study does not include direct evidence that the underlying circuits were reorganized - only that population-level signals become more discriminable. As such, the term "plasticity" may overstate the conclusions and should be interpreted with caution unless validated by additional causal or longitudinal data.

Thank you for your important comment. We agree that the term "plasticity" may overstate our conclusions, as our study focuses on population-level signal changes rather than direct evidence of circuit-level reorganization.

To address this, we have revised the manuscript to clarify that while the observed changes in neural separability suggest functional reorganization of sensory representations, they do not confirm structural plasticity. We have updated the wording throughout the manuscript to emphasize that these findings reflect functional reorganization in response to short-term visual input loss, rather than structural or long-term plasticity.

We also updated the discussion to highlight the need for future research with more invasive approaches to validate the causal mechanisms behind these rapid changes in neural dynamics.

(4) The study highlights the forelimb region of S1 and a post-contact temporal window as particularly important for decoding texture, based on occlusion and integrated gradient analyses. However, this finding may be somewhat circular: The LFPs were aligned to forelimb contact, and the floor textures were sensed primarily via the forelimbs, making it unsurprising that forelimb electrodes were most informative. The observed temporal window corresponds directly to the event-aligned epoch, and while it may shift slightly in duration in the dark, this could reflect general differences in sensory gain or arousal, rather than changes in stimulus-specific encoding. Thus, while these findings are consistent with somatotopy and context-dependent dynamics, they do not provide strong independent evidence for novel spatial or temporal organization.

Thank you for your insightful comment. We understand your concern that the finding of forelimb electrodes being most informative might seem circular, given that the LFPs were aligned to forelimb contact, and the floor textures were primarily sensed by the forelimbs. This design choice was intentional, as the task focused on texture perception through the forelimb, and the forelimb subregion of S1 is naturally expected to play a dominant role in this process. While this somatotopic specificity may make the results predictable, our aim was to emphasize the changes in temporal dynamics of neural processing under visual deprivation.

We observed a shift in the temporal window's duration in the dark condition, which we interpret as a change in how texture information is processed without visual input. While this could reflect sensory gain or arousal differences, the lack of significant differences in locomotor speed or other behavioral measures (Supplementary Figure 1) suggests that these changes are more likely due to functional reorganization of sensory processing.

We have clarified in the discussion that the shift in the temporal window is consistent with previous research on sensory reorganization involving both spatial and temporal cortical adjustments. While we do not claim novel spatial or temporal organization, we emphasize that the shift in temporal dynamics suggests adaptation in encoding strategy for texture perception in the absence of visual input. Future studies measuring arousal states (e.g., pupil diameter or cortical state markers) would help distinguish the contributions of arousal versus sensory reorganization to these dynamics.

(5) While the neural data suggest enhanced tactile representations, the study does not assess whether rats' actual tactile perception improved. Without a behavioral readout (e.g., discrimination accuracy), claims about perceptual enhancement remain speculative.

Thank you for raising this important point. We agree that while the neural data suggest enhanced separability of tactile representations in the dark condition, we do not directly assess whether these changes translate into improved tactile perception behaviorally.

However, the primary aim of our study is not to claim perceptual enhancement, but to demonstrate that neural representations in the somatosensory cortex can rapidly reorganize in response to visual deprivation. To clarify this distinction, we have revised the manuscript to emphasize that the observed neural changes in S1 are consistent with functional reorganization of tactile representations, rather than a direct indication of perceptual improvement.

Future studies will be crucial to directly test whether the enhanced separability of tactile representations in S1 correlates with improved tactile perception in a behavioral task. We have highlighted this as an avenue for future research to better understand the link between neural changes and perceptual outcomes.

(6) In addition to point 4, the authors discuss implications for sensory rehabilitation, including Braille training and haptic feedback enhancement. However, the lack of actual chronic or even more acute pathological sensory deprivation, behavioral data, or subsequent intervention in this study limits the ability to draw translational conclusions. It remains unknown whether the more distinct neural representations observed actually translate into better tactile performance, discriminability, or perception. Additionally, extrapolating from rats walking on sandpaper in the dark to human rehabilitative contexts is speculative without a clearer behavioral or mechanistic bridge. The potential is certainly there, but the claim is currently aspirational rather than empirically grounded.

Thank you for raising this important point. Upon careful consideration, we have decided to remove the discussion of sensory rehabilitation implications from the revised manuscript. We have refocused the manuscript to concentrate solely on the neural findings related to tactile encoding reorganization in response to short-term sensory deprivation, avoiding speculative extrapolation to human rehabilitative contexts. This revised approach ensures that the manuscript emphasizes the empirical findings without overstating the translational potential.

(7) While the CNN showed good performance, details on generalization robustness and validation (e.g., cross-validation folds, variance across animals) are not deeply discussed. Also, while explainability tools were used, interpretability of CNNs remains limited, and more transparent models (e.g., linear classifiers or dimensionality reduction) could offer complementary insights.

We appreciate the reviewer’s valuable feedback. In response to the concern about generalization robustness and validation, we have now conducted 5-fold cross-validation to assess the model's performance within animals (Figure 6C). We also have added supplementary information on the average silhouette scores across the different folds and animals (Supplementary Table 1, 2). These details are provided in the methods section and discussed in the results to offer a clearer picture of the model's robustness and consistency across rats.

Regarding the interpretability of CNNs, we acknowledge that deep learning models can lack transparency. We also attempted classification using more transparent models such as PCA and SVM, but their performance did not exceed chance level (Supplementary Figure 2). This indicates that while these simpler models are more interpretable, they cannot capture the complex representations in the LFPs, making deep learning models like CNNs necessary for extracting these insights.

**Reviewer #2 (Public review):**

(1) Despite applying explainability techniques to the CNN-based decoder, the study does not clearly demonstrate the precise "subtle, high-dimensional patterns" exploited by the CNN for surface roughness decoding, limiting the physiological interpretability of the results. Additional analyses (e.g., detailed waveform morphology analysis on grand averages, time-frequency decompositions, or further use of explainability methods) are necessary to clarify the exact nature of the discriminative activity features enabling the CNN to decode surface roughness and how these change with the sensory context (i.e., in light or darkness).

Thank you for your insightful comment. We recognize the importance of clarifying the exact nature of the high-dimensional neural patterns that the CNN exploits for surface roughness decoding. In response, we have performed additional analyses to provide a more detailed explanation of the CNN's decision-making process and the discriminative features it learned:

Grand-Average LFP Waveforms Analysis: We calculated the grand-average LFP waveforms for each texture × lighting condition (Figure 4A). While visual inspection did not reveal distinct features in the averaged waveforms, we explored the channel-wise correlations between textures under both light and dark conditions (Figure 4B). We found that the correlation between textures was lower in the dark condition, suggesting that LFPs become more distinct between textures when visual input is absent, which aligns with the CNN’s output.

Time-Frequency Decomposition (Wavelet Analysis): We also performed time-frequency decomposition of the LFPs using wavelet transforms (Figure 4D). No prominent differences emerged across texture × lighting conditions in the spectral domain. However, upon computing differences in wavelet features between light and dark conditions and analyzing the relationship with the CNN's attribution scores (Supplementary Figures 5A-C), we observed a negative correlation in the 50-60 Hz range and a positive correlation in the 80-90 Hz range. This suggests frequency-specific modulation in LFP activity that may contribute to texture representations, providing further support for the CNN’s learned features.

(2) The claim regarding cross-modal representation reorganization heavily relies on a silhouette analysis (Figure 5C), which shows a modest effect size and borderline statistical significance (p≈0.05 with n=9+2). More rigorous statistical quantification, such as permutation tests and reporting underlying cluster distances for all animals, would strengthen confidence in this finding.

Thank you for your thoughtful comment. We appreciate your suggestion to strengthen the statistical rigor of our analysis regarding the cross-modal representation reorganization. In response, we have implemented several additional analyses to more rigorously quantify the separability of neural representations between light and dark conditions:

(1) Permutation Test for Cluster Separability: We performed a permutation test to assess whether the observed differences in cluster separability between light and dark conditions were statistically significant or could have arisen by chance. The results showed that the silhouette scores for the dark condition consistently exceeded the 95th percentile of the null distribution (Supplementary Figure 4). This permutation test strengthens the validity of our findings, indicating that the enhanced separability in darkness is a systematic reorganization of neural representations, not due to random fluctuations.

(2) Reporting Cluster Distances: To address concerns about the modest effect size and borderline significance, we have explicitly reported the underlying cluster distances in the form of silhouette scores for each individual animal (Supplementary Table 1, 2). These values reflect the Euclidean distance between clusters within each rat, providing a clearer understanding of the separability observed.

(3) Additional Statistical Analysis on Silhouette Scores: To further enhance the rigor of our statistical analysis, we recalculated the silhouette scores using 5-fold cross-validation within each animal, ensuring that our results are robust across multiple data splits (Figure 6C).

By incorporating these additional analyses and reporting detailed cluster distances, we believe we have significantly strengthened the confidence in our claim of cross-modal reorganization.

(3) While the authors recorded in the somatosensory cortex, primarily known for its tactile responsivity, I would be cautious not to rule out a priori the presence of crossmodal (visual) responses in the area. In this case, the stronger texture separation in darkness might be explained by the absence of some visually-evoked potentials (VEPs) rather than genuine cross-modal reorganization. Clarification is needed to rule out visual interference and this would strengthen the claim.

Thank you for raising this important point. In response to your concern, we carefully examined whether visually-evoked potentials (VEPs) could be present in the S1 recordings, particularly under the light condition. However, we observed that this experiment did not involve any cue-guided visual stimulation, such as flashing lights or visual cues aligned with the LFP recordings. Without such external visual stimuli, it is unlikely that VEPs would be reliably evoked in the S1. Therefore, we believe the stronger texture separation observed in the dark condition is not due to visual interference, but rather reflects a genuine sensory reorganization in response to the absence of visual input.

(4) Behavioural controls are limited to gross gait parameters; more detailed analyses of locomotor behavior and additional metrics (e.g., pupil size or locomotor variance) would robustly rule out potential arousal or motor confounds.

Thank you for your insightful comment regarding behavioral controls. In response, we have added locomotor speed traces aligned with corresponding LFPs (Supplementary Figure 1) to demonstrate that locomotion remained consistent across trials, irrespective of environmental condition (light vs. dark). Additionally, we report locomotor speed variance over 10-minute blocks to confirm no significant motor changes affecting neural recordings. These analyses indicate that LFP differences are unlikely due to locomotor confounds.

While measuring pupil size could be useful for assessing arousal, the camera resolution in our study was insufficient for reliable measurements. We have noted this limitation in the Discussion and recommend that future studies with high-resolution eye-tracking explore arousal's role in sensory processing in S1.

(5) The consistent ordering of trials (10 minutes of light then 10 minutes of dark) could introduce confounds such as fatigue or satiation (and also related arousal state), which should be controlled by analyzing sessions with reversed condition ordering.

Thank you for highlighting the potential confounds due to trial ordering. To address this, we reversed the condition order (dark before light) in a subset of sessions from six rats and reanalyzed the data (Supplementary Figure 3). The results showed not significant, but increase separability in the dark condition, suggesting that the enhanced separability in the dark condition is not due to trial order effects like fatigue or satiation. While order effects may contribute to trial-to-trial variability, the consistent pattern of enhanced separability in the dark further supports the interpretation that visual deprivation directly influences the reorganization of tactile representations in S1.

(6) The focus on forelimb-aligned LFP analyses raises the possibility that hindlimb-aligned data might yield different conclusions, suggesting alignment effects might bias the results.

Thank you for your insightful comment on the potential bias of forelimb-aligned LFP analyses. We acknowledge that the choice of alignment event can influence the results and appreciate the suggestion to consider hindlimb-aligned data. However, our experimental design specifically focused on forelimb S1. The forelimb region of S1 was oversampled in our array, and as expected, we observed larger responses there, consistent with the known somatotopic organization of S1.

While hindlimb-aligned data could provide additional insights, it is not directly relevant to the primary question of how forelimb S1 codes tactile information under visual deprivation. We do not believe the forelimb alignment introduces a bias, as it aligns with the sensory task being investigated. However, we recognize the value of exploring alternative alignments and have now included a discussion in the Methods section regarding the rationale for our design choices.

(7) The authors' dismissal of amplitude-based metrics as ineffective is inadequately substantiated. A clearer demonstration (e.g., event-related waveforms averaged by conditions, presented both spatially and temporally) would support this claim.

Thank you for your constructive comment. In response, we have added a more detailed analysis of event-related waveforms, averaged across conditions (light vs. dark, smooth vs. rough textures), and presented them spatially and temporally aligned to forelimb contact (Figure 4A). These waveforms did not show clear, distinct features that could differentiate conditions, which highlights the limitations of traditional amplitude-based metrics in detecting subtle neural activity changes related to visual deprivation.

We further performed channel-wise correlation analyses (Figure 4B), revealing stronger texture correlations in the light condition, indicating that averaged waveforms do not capture the nuanced differences in neural dynamics. Additionally, time-frequency spectrograms and channel–channel correlation matrices (Figures 4C and 4D) did not show distinct condition differences, reinforcing the limitations of amplitude-based metrics.

These findings, along with the superior performance of machine learning-based decoding methods (e.g., CNN), support our claim that amplitude-based approaches are insufficient for fully capturing the complexity of the neural data.

(8) Wording ambiguity regarding "attribution score" versus "activation amplitude" (Figure 5) complicates the interpretation of key findings. This distinction must be clarified for proper assessment of the results.

Thank you for pointing out the ambiguity between "attribution score" and "activation amplitude." To address this, we have revised the manuscript to use "attribution score" only.

(9) Generalization across animals remains unaddressed. The current within-subject decoding setup limits conclusions regarding shared neural representations across individuals. Adopting cross-validation strategies and exploring between-animal analyses would add significant value to the manuscript.

Thank you for highlighting the importance of generalization across animals. While our study focused on within-subject decoding, we acknowledge that this limits conclusions about shared neural representations across individuals. We expect that inter-animal generalization would be challenging, as models trained on data from a single rat may not perform well on data from others due to differences in electrode placement, brain anatomy, and neural representations. We recognize the value of cross-validation strategies and between-animal analyses and will consider them in future work to address this limitation.

**Recommendations for the authors:**

**Reviewer #1 (Recommendations for the authors):**

(1) I would strongly recommend that the authors refine their introduction to be more concise. Many concepts and study aims are repeated many times and, therefore, present as highly redundant text. The introduction may be half the length and still contain the important concepts to set up the justification for the study. I would also suggest refining to be less about sensory deprivation (e.g., with blindness) and more in relation to context, as the acute nature of the study allows one to conclude more about the latter than the former.

Thank you for your feedback on the introduction. We have revised the section to reduce redundancy and present the key concepts more concisely. We also streamlined the study aims and focused more on the context of the acute nature of the study, as you suggested, rather than emphasizing sensory deprivation. This revision better aligns with the main focus of the research and improves clarity. We believe the updated introduction provides a more direct justification for the study.

(2) I am not sure if Figures 1-3 are meant to be in grey-scale for some reason (perhaps to represent light and dark), but I would encourage the authors to examine if this is necessary, as the use of color generally helps one more easily follow Figures.

Thank you for this suggestion. Upon review, we agree that the use of color would enhance the clarity and readability of our figures. We have revised the figures including the newly added supplementary figures to incorporate color.

(3) Figure 5, Figure legend title - check wording.

Thank you for pointing this out. The title has been adjusted for consistency with the other figure legends.

**Reviewer #2 (Recommendations for the authors):**

(1) Analyses that would strengthen the main claims (major):

(a) Identify the features exploited by the CNN.

(i) Provide grand-average LFP waveforms for each texture × lighting condition (fore- and hind-limb channels shown separately, spatially arranged as in Figure 3C) and try to relate them to the decoding strategy learned by the CNN.

Thank you for your helpful suggestion. We have calculated the grand-average LFP waveforms for each texture × lighting condition and included them in Figure 4A, with fore- and hind-limb channels shown separately and spatially arranged as in Figure 3C. Upon visual inspection, the mean waveforms did not reveal clear, distinct features. To further investigate, we computed the channel-wise correlation between different textures under both dark and light conditions. By subtracting the correlation coefficients for the dark environment from those in the light, we observed that the correlation between textures was lower in the dark environment (Figure 4B). This suggests that LFPs are more distinct between textures in the dark, supporting the CNN model's output. However, this also indicates that the CNN has captured more complex, nuanced information, as it is able to discriminate between LFPs on a single-trial basis, rather than relying on mean traces.

To assess how the correlation between average LFP waveforms varied across channels, we also calculated the channel-channel correlation matrix for all 32 channels in each condition. While we found stronger correlations within each S1 subregion, we did not observe clear differences of correlation matrix between light and dark conditions, nor between different textures (Figure 4C).

(ii) Add channel-wise and time-frequency maps (e.g., wavelet or spectrograms) for each texture × lighting condition and try to relate them to the decoding strategy learned by the CNN.

Thank you for the valuable suggestion. We calculated wavelet features for each LFP segment and averaged them across trials to assess differences in LFP between light and dark conditions, as well as across textures (Figure 4D). However, no distinct differences were observed in the spectral map. To investigate further, we computed the differences in spectral maps for LFPs in light and dark trials. We then calculated the difference in attribution scores derived from the integrated gradient map (Supplementary Figure 4A). Subsequently, we calculated the correlation coefficients between the differences in integrated gradients and the differences in power across each frequency band in the spectral map (Supplementary Figures 4B and 4C). A negative correlation was found in the 50-60 Hz range, while a positive correlation was observed in the 80-90 Hz range. These findings suggest that frequency-specific patterns of LFP activity in different conditions may be linked to the texture representations captured by the CNN model. We have included a discussion of these findings in [lines 463-468].

(b) Quantify the "enhanced separability in darkness" more rigorously.

(i) Report cluster-distances (e.g. Euclidean) for each individual animal.

We thank the reviewer for this helpful comment. When calculating the silhouette score, we used Euclidean distance as the distance metric. The silhouette score is defined for each data point as the difference between the average distance to points within its assigned cluster and the average distance to points in the nearest other cluster, normalized by the larger of the two values. Thus, the silhouette score inherently reflects the relative cluster distances both within and across conditions for each individual animal. Because we report and statistically analyze silhouette scores (Figure 6C), these values already quantify and compare the Euclidean cluster distances across conditions at the animal level. For clarity, we have now added a definition of the silhouette score in the Methods section of the main text [lines 269-278]. We also included the calculated silhouette scores in Supplementary Table 1.

(ii) Run a permutation or bootstrap test (shuffling darkness/light labels within animals) to obtain an empirical null distribution for cluster separability in the network embedding space.

We thank the reviewer for this important suggestion. In response, we implemented a permutation test to assess the robustness of our cluster separability results. Specifically, we shuffled the darkness/light labels within each animal and recalculated silhouette scores across 1000 resamples to generate an empirical null distribution. The observed separability between light and dark conditions consistently exceeded the 95th percentile of the null distribution (Supplementary Figure 3). This confirms that the enhanced cluster separability in darkness was not attributable to random fluctuations in labeling but instead reflected a systematic reorganization of neural representations.

(c) Control for possible visually-evoked potentials (VEPs).

(i) Search the LFPs recorded in light for stereotyped VEP components and/or comment on this possible confound (i.e., VEPs in S1?).

Thank you for raising this point. Although it would be interesting to observe if a VEP is present in the S1 of rats, this experiment did not involve cue-guided visual stimulation. Additionally, there was no environmental visual cue that could serve as an external trigger to align the LFPs for VEP analysis in S1. Furthermore, since even the somatosensory evoked potential was not clearly visible in the S1 LFP without averaging the aligned LFPs, it is unlikely that we would be able to observe VEPs in single trials.

(d) Address behavioral and arousal confounds.

(i) Provide example locomotor-speed traces (aligned with corresponding LFPs) and report locomotor-speed variance across the 10-min blocks.

Thank you for your comment. We had speedometer installed for the recording of the last two rats. We have now provided example speed traces and the speed variance across blocks in Supplementary Figure 1. The traces show that the locomotor-speed was stable in each trial.

(ii) If available from the camera recordings, include pupil diameter as a proxy for arousal; otherwise, discuss explicitly how arousal changes might affect S1 LFPs.

Thank you for this suggestion. We strongly agree that measuring pupil diameters should be incorporated into future studies. However, because our camera did not have sufficient resolution to capture pupil diameters, we have addressed this limitation in the discussion section [lines 525-537].

(e) Address order effects (and motivation/satiety confounds)

(i) Present at least a subset of sessions in which the dark block precedes the light block; re-analyze the silhouette score/discriminability with block order as a factor.

Thank you for this helpful suggestion. We conducted additional analyses using sessions from 6 rats in which the dark block preceded the light block (Supplementary Figure 5A). Using the same model architecture, we calculated the silhouette score for each rat (Supplementary Figure 5B). However, when the order was reversed (dark preceding light), this discriminability effect disappeared. Thus, while we observed a trend toward higher scores in the dark condition, no statistically significant differences in texture discriminability were observed.

If trial order alone accounted for the increase in discriminability, reversing the order would be expected to yield higher silhouette scores in the light condition. Our findings suggest that factors related to order (e.g., thirst or motivation, as you proposed) are not the sole contributors. Furthermore, previous studies in human participants have shown that brief blindfolding can produce lingering increases in tactile sensitivity, indicating a lasting effect of visual deprivation. Thus, the absence of significant differences in texture representation when the dark condition preceded the light condition may reflect such lasting effects. We have included a discussion in [lines 441-452].

(ii) Discuss explicitly the potential confounding effect of motivational state/thirst.

We appreciate the reviewer’s insightful comment. In the revised manuscript, we now explicitly address the potential confounding role of motivational state and thirst in shaping our results. Because animals were water-restricted to maintain task engagement, it is possible that increasing thirst or fluctuating motivation over the course of a session could alter arousal or attentional state, thereby influencing neural separability. However, when the trial order was reversed (dark condition preceding light), silhouette scores did not show a significant increase in the second (light) trial. Thus, while we acknowledge that motivational state may contribute to trial-to-trial variability, the systematic increase in separability during darkness cannot be fully explained by thirst or motivational confounds. This addition has been incorporated into the discussion section [lines 441-452].

(f) Alignment control and the role of forelimb S1.

(i) Repeat the decoding analysis with LFPs aligned to hind-limb strike; report whether the fore-limb dominance persists.

Thank you for your thoughtful suggestion. We appreciate the opportunity to clarify. Our study was designed to ask a different question: how the absence of visual input reorganizes tactile encoding for the body part that actually initiates texture contact in our paradigm (the forepaw). Accordingly, all analyses were aligned to forelimb strike and our array intentionally oversampled S1-forelimb relative to S1-hindlimb (18 vs. 14 electrodes; Fig. 1F–G), yielding clear topographic forelimb-locked event-related responses (Fig. 3B–D) and forelimb-channel dominance in the decoding explainability analyses (Fig. 5D–E). Repeating the full decoding locked to hind-limb strike would test a different hypothesis and would be difficult to interpret for three reasons:

Design/measurement alignment. Our kinematic detection was built to identify forelimb foot strikes. Extending the detector to hindlimb would require new model training/validation and introduces uncertainty in the exact contact timing relative to the LFP segments we analyze.

Sampling asymmetry. The array and cortical magnification are not balanced across subregions (18 forelimb vs. 14 hindlimb electrodes; Fig. 1G), so a hind-limb–aligned comparison would be confounded by unequal coverage and signal-to-noise across S1 subdivisions rather than reflecting true “dominance.”

Scope of the claim. We do not claim that the forelimb is globally more informative about texture; we show the intuitive and topographically specific result that “forelimb S1 codes textures touching the forelimb,” and that these representations become more separable in darkness (silhouette increase; Fig. 5C). A hind-limb–locked re-analysis would likely reveal hindlimb contributions when the hindpaw is the alignment event — but that would not change the central conclusion about darkness enhancing tactile representational separability.

To address the underlying concern about generality without introducing the above confounds, we have clarified these design choices and limitations in the revised Methods [lines 194-197].

(g) Amplitude-based baseline.

(i) Show that a simple linear discriminant or logistic-regression model on peak amplitudes (and/or other simple features like trough width/slope) cannot reach the CNN's accuracy. This kind of "baseline" analysis could also be useful to pinpoint the discriminative features learned by the CNN.

Thank you for your insightful suggestion. We agree that performing a baseline comparison with a simpler model could help highlight the advantage of using a CNN. However, in our dataset, individual LFP traces do not exhibit clear peaks or well-defined features such as peak amplitude, width, or energy, which makes feature extraction using traditional methods like linear discriminants or logistic regression challenging.

To address this, we performed principal component analysis (PCA) on the raw LFP traces to reduce the dimensionality and applied a support vector machine (SVM) classifier on the reduced features, in line with the approach used for the CNN models (Supplementary Figure 2A). The results of this analysis, demonstrate that the SVM model struggles to effectively discriminate between conditions, further reinforcing the necessity of the CNN model. The CNN’s ability to automatically learn complex features from the raw LFP data appears to be a crucial factor in achieving superior classification performance (Supplementary Figure 2B).

(h) Cross-validation and inter-animal generalization.

(i) Consider replacing the single 80/20 split with k-fold cross-validation within animals.

Thank you for this suggestion. Instead of using an 80/20 split, we performed 5-fold cross-validation on all rats. The silhouette scores were averaged within each animal across the five folds, and Figure 6C was updated accordingly. After performing a paired t-test, we still observed a significant difference in silhouette scores between the light and dark conditions.

(ii) Comment on inter-animal generalization.

Thank you for this valuable feedback. Although we did not explicitly test inter-animal generalization, it is unlikely that a model trained on data from one rat would perform equally well when classifying data recorded from another animal. This limitation arises from two main factors. First, despite careful efforts to implant electrodes in the same brain region and cortical layer across experiments, it is impossible to align all 32 electrodes to identical coordinates. Consequently, the recorded LFPs are obtained from slightly different locations, which may reflect distinct neural processing. Second, even within the same species, individual animals differ in brain size and neural circuit organization. Thus, even if electrodes could be placed at identical anatomical locations, inter-individual variability in brain structure would still lead to differences in the recorded signals. Because deep learning models are often sensitive to small perturbations in their input data, we believe that robust inter-animal generalization is unlikely without fine-tuning the model using data from the target animal. This comment has been inserted in the Discussion [lines 494-507].

(2) Writing, figure and terminology improvements (minor):

(a) Figure 5F-G axis label. Decide on either "attribution score" or "activation amplitude" and use that term consistently in panels, legend, and text (currently, I believe it could be confused with raw signal amplitude).

We have unified the terminology to "attribution score" and applied this consistently across the panels, legend, and text.

(b) Throughout the manuscript, use "population-level activity" or "average population dynamics" when discussing LFPs (I believe it is more correct to reserve "population code" for multiple single-unit datasets).

We agree with the reviewer’s point and have adapted the term "population dynamics" to describe LFP information consistently throughout the manuscript.

(c) Lines 219-221, state down-sampling to 2 kHz, whereas line 289 mentions 10 kHz. Reconcile these numbers.

We apologize for the confusion and thank the reviewer for thoroughly reading the manuscript. Our original sampling rate was 30 kHz, and all analyses were performed on data resampled to 10 kHz. The reference to 2 kHz was an error, and we have corrected it.

(d) Specify the tail of each statistical test mentioned in the manuscript and any multiple-comparison correction used.

We have specified the tail of each statistical test and any multiple-comparison corrections used in the "Data Analysis" section of the Methods.

(e) Line 244: "variables (He et al., 2015)" → "variables (He et al., 2015)".

We have corrected this formatting issue and revised it to "variables (He et al., 2015)".

(f) Line 253: "one-dimentional" → "one-dimensional".

We have corrected the spelling error and revised it to "one-dimensional".

(3) Data and code sharing:

(a) Consider depositing data and code for the analysis in public open repositories.

Thank you for your suggestion. We have set up a public GitHub repository to share the code. Since the full dataset is quite large (~400GB), we have uploaded a smaller example dataset for the analysis.
